# Capturing a Comprehensive Picture of Biological Events From Adverse Outcome Pathways in the Drug Exposome

**DOI:** 10.3389/fpubh.2021.763962

**Published:** 2021-12-17

**Authors:** Qier Wu, Youcef Bagdad, Olivier Taboureau, Karine Audouze

**Affiliations:** ^1^INSERM U1124, CNRS ERL3649, Université de Paris, Paris, France; ^2^INSERM U1133, CNRS UMR8251, Université de Paris, Paris, France

**Keywords:** network science, AOP, bipartite network, drug-AOP, AON, NAM

## Abstract

**Background:** The chemical part of the exposome, including drugs, may explain the increase of health effects with outcomes such as infertility, allergies, metabolic disorders, which cannot be only explained by the genetic changes. To better understand how drug exposure can impact human health, the concepts of adverse outcome pathways (AOPs) and AOP networks (AONs), which are representations of causally linked events at different biological levels leading to adverse health, could be used for drug safety assessment.

**Methods:** To explore the action of drugs across multiple scales of the biological organization, we investigated the use of a network-based approach in the known AOP space. Considering the drugs and their associations to biological events, such as molecular initiating event and key event, a bipartite network was developed. This bipartite network was projected into a monopartite network capturing the event–event linkages. Nevertheless, such transformation of a bipartite network to a monopartite network had a huge risk of information loss. A way to solve this problem is to quantify the network reduction. We calculated two scoring systems, one measuring the uncertainty and a second one describing the loss of coverage on the developed event–event network to better investigate events from AOPs linked to drugs.

**Results:** This AON analysis allowed us to identify biological events that are highly connected to drugs, such as events involving nuclear receptors (ER, AR, and PXR/SXR). Furthermore, we observed that the number of events involved in a linkage pattern with drugs is a key factor that influences information loss during monopartite network projection. Such scores have the potential to quantify the uncertainty of an event involved in an AON, and could be valuable for the weight of evidence assessment of AOPs. A case study related to infertility, more specifically to “decrease, male agenital distance” is presented.

**Conclusion:** This study highlights that computational approaches based on network science may help to understand the complexity of drug health effects, with the aim to support drug safety assessment.

## Introduction

Nowadays, it is established that the increase of civilization diseases such as obesity or type 2 diabetes is not only explained by genetic changes in individuals, but may also be due to exposure to environmental factors. The concept of the exposome was proposed by Wild (1) and refined by Rappaport (2), which refers to the totality of the environmental exposures an individual is exposed to, during their entire lifetime from conception until death. The exposome aims at capturing all non-genetic factors such as physical stressors, biologicals, psychological, and social stresses, as well as chemical exposure. The exposome is therefore complementary to the genome, and the integration of both, representing the exposome-genome paradigm will be very useful to identify predictive markers for disease prevention at the population level (3). Humans are daily exposed to a high number of hazardous chemicals (drugs, toxicants, pollutants, and nutrients) from various sources such as cosmetics, diet, or medical treatments. The potential effect of the chemical exposome may contribute to disease risks and adverse outcomes, as it has been established in various biomonitoring and epidemiological studies from European projects (e.g., HELIX, HEALS, and HBM4EU) (4–6).

Even if drugs have been primarily designed to treat specific diseases, they may also influence health effects by displaying adverse effects. For example, aspirin and paracetamol are the common drugs to treat pain and fever. However, both drugs can lead to hepatoxicity with high doses (7). Analgesic use has also been associated with endocrine and reproductive effects (8). Many drugs interact with multiple biological targets, as referred to the polypharmacy action of drugs, and these unintended actions could cause adverse effects (9). Polypharmacy remains one of the major challenges in drug development as the modes of action of many drugs are not completely understood. To overcome this limitation, network pharmacology, which considers the drug action across multiple biological layers, is a promising approach (10). A recent review investigated the drug–exposome interaction, which shows how drugs can interact with other chemicals to which humans are exposed, and therefore how they may impact and influence human health (11). For example, chemotherapeutics can negatively be impacted by drug-exposome interactions, resulting in less drug efficacy, an increase of drug resistance, and adverse effects.

Emerging technological advances (e.g., high throughput screening (HTS) screening, omics) have supported the generation of the high number of data, allowing the development of computational studies, at cells and organs levels, to investigate biological mechanisms and to predict potential adverse effects of chemicals (including drugs). It is now possible to develop models based on omics data obtained for drugs or other toxicants (12). Therefore, exposomics can help in identifying novel chemical exposure-health associations, which create opportunities to prioritize relevant chemicals for risk assessment (13). Several systems toxicology models were developed based on network science to assess the chemical exposome effects on human health, some being applied to predict the effects of endocrine disruptors or persistent organic pollutants (14–18).

As phenotypes are the results of the complex interplay between chemicals and genetic factors, understanding the interactions between chemicals and diseases is essential. Recently, the tool “phexpo” was designed to explore bidirectional chemical and phenotype interactions (19). Also, the HExpoChem webserver allows exploring and predicting human health risks from various chemical exposure sources (20). All these integrative and computational approaches are complementary to existing approaches (pharmacogenomics), and combined together could be very useful for drug assessment. Yet, there is still a need to improve our understanding of the adverse effects of drugs in humans and the biological pathways they may perturbto fully assess their real impacts on humans.

Recently, a new concept has emerged in the field of toxicology, which is adverse outcome pathways (AOP) (21). AOPs are linear representations of biological perturbations [molecular initiating events (MIEs) and key events (KEs)] at different levels of the biological organization caused by a stressor (that could be a chemical), which lead to an adverse outcome (AO). The Organization for Economic Cooperation and Development (OECD) has classified MIEs and AOs as two specific KEs in AOPs. When evidences exist, KEs are linked together through Key Event Relationships (KERs), that are the causal linkages between two events. Even if AOPs are chemical agnostics, they are recognized as a tool for risk assessment of chemical effects to better understand their modes of action, and therefore could be also translated to the drug exposome. KEs are not uniquely associated with one AOP but can be shared between several AOPs, leading therefore to AOP networks (AONs) that reflect the real complexity of biological systems (22).

Using the concept of AOP, we proposed a systems toxicological approach to investigate, in a systematic way, linkages between drugs and biological events (MIE, KE, and AO) with the development of a network-based model. First, a bipartite network was generated by integrating known drug–event associations extracted from various data sources. This bipartite network was then transformed into an event–event monopartite network to explore the AOP space of drugs. A recently published method measuring the uncertainty and information loss of network was applied to assess the conservation of the information from the bipartite to the monopartite network (22), and the obtained results in our study are discussed below.

## Materials and Methods

**Figure 1:**
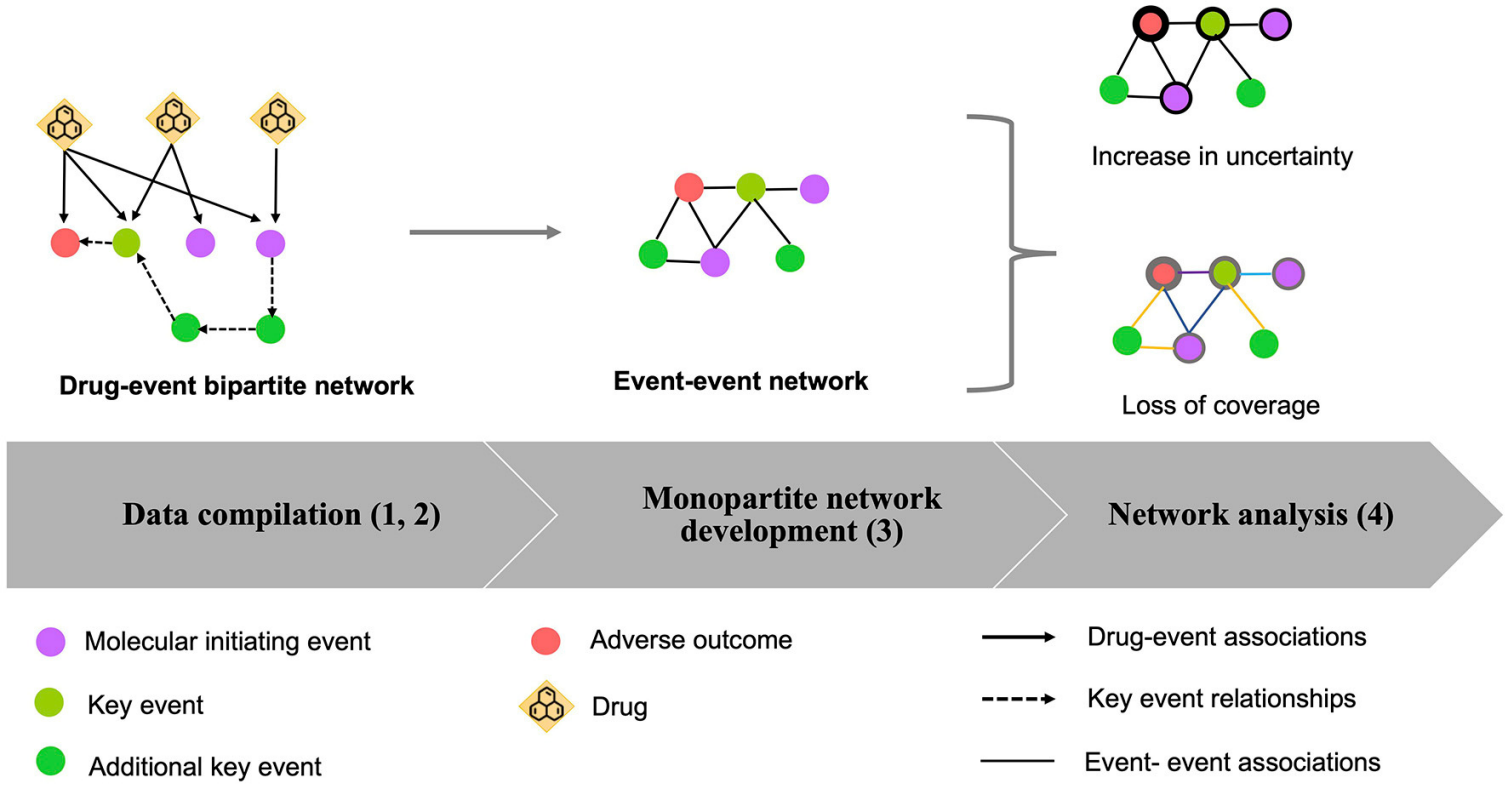
Workflow of the procedure to assess AOPs in the drug exposome. A multistep procedure was developed: (1) Data compilation: drug–event associations were extracted from various data sources (solid lines). Several event types were considered, which are molecular initiating events (MIEs) (purple circles), key events (KEs) (light green circles), and adverse outcomes (AOs) (red circles); (2) Bipartite network development: a bipartite drug-event network was created based on the compiled data. To enrich the created network, KE relationships (KERs) (dash lines) extracted from the AOP-wiki database were identified, allowing to add other KEs (green circles); (3) Generation of the monopartite network: if two events shared at least one drug in the bipartite network, a link was created between these two events in the monopartite network. The transformation of the bipartite network to a monopartite network was done by using the “guilt by association” principle; (4) Network analysis: the loss of information from the bipartite network to the monopartite network was quantified by calculating the “increase of uncertainty” and the “loss of coverage.”

The developed systems biology approach, to explore the drug effects in the biological space of AOPs, is a multistep procedure. A workflow of the proposed strategy is illustrated in Figure 1.

### Data Set

Drug-event associations were extracted from two complementary datasets: the AOP-wiki database (https://aopwiki.org/, as of August 2020) (23) and the U.S. Environmental Protection Agency web-based CompTox Chemistry dashboard (https://comptox.epa.gov/dashboard/, as of March 2021) (24). The AOP-wiki database is a data source developed by the OECD. From this data source, 316 AOPs and 1,393 relevant events (i.e., MIE, KE, and AO) that are known to be linked to 615 stressors were extracted. All defined AOPs were taken into consideration, even if they are not yet validated by the OECD. The CompTox database contains a wide range of data related to chemical toxicity including, AOP information, human exposure, and other kwnoledge for over 88,300 chemicals. Direct and putative associations between 22,038 chemicals and 103 AOPs are stored in this database (25). As both databases provided information related to various stressor types (drugs, pollutants, etc) we matched the drug names and CAS numbers obtained from the DrugBank (26) and the DrugCentral databases (27) to be able to keep only information related to drugs, without loosing or duplicating knowledge. Consequently, we were able to extract unique drug-event and drug-AOP associations from these two data sources (Comptox and AOP-wiki).

To enrich our list of drug-AOP associations, an additional manual curation of the literature using the PubMed database (https://pubmed.ncbi.nlm.nih.gov/) (as of September 2020) was performed. The searches were done using the exact terms of interest for which we were missing links from the previous data integration step. As an example, cooccurence between ciprofibrate and activation, PPAR alpha was investigated, and identified publications were fully read for validation.

### Development of the Drug-Event Bipartite Network

A drug-event bipartite network was created using the R software (version 3.5.23) based on the previously compiled data from the different data sources. Each node in the network represents either a drug or an event (MIE, KE, or AO). To enrich the connections between drugs and events, and to have a more comprehensive mapping of the drugs in the AOP space, KERs were investigated using the AOP-wiki database (as of September 2020). Globally, if a KER exists between two events (MIE, KE, AO) that are present in the bipartite network, then the biological events linking this KER was selected and included to the network.

### Creation of the Monopartite Network of Events in the Drug Space

To analyze the relationships of events targeted by drugs, the bipartite network was projected into a monopartite network using the guilt by association principle (28), i.e., linking events through the shared connections between drugs, MIE, KE, and AO. With this projection, some information might be lost. Therefore, some scores measuring the uncertainty and the loss of coverage of the monopartite network were developed. For better visualization of the obtained complex networks, we exported both networks (bipartite and monopartite) to Cytoscape V3.7.2 which is an open-source library for network analysis and visualization (29). This tool allows displaying of an interactive representation of the drug–event and event–event networks.

### Analysis of the AOP Monopartite Network

**Figure 2:**
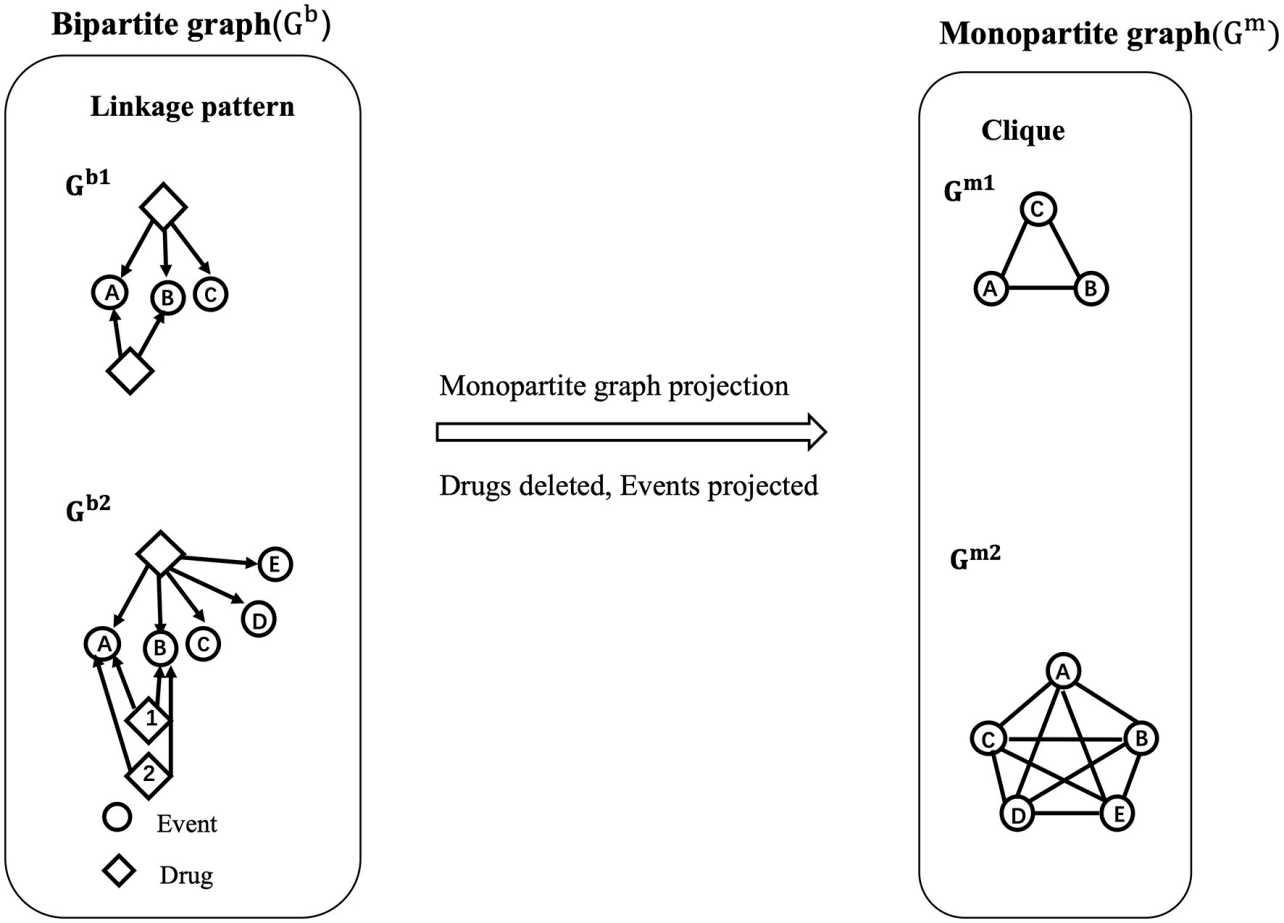
Illustration of bipartite network projection to a monopartite network. In a bipartite network G^b^, containing two types of nodes (events represented as circles and drugs as diamonds), the associations between drugs and events are defined as “linkage patterns”. For example, in G^b1^, the A, B, C events are connected to a drug 1 *via* 3 edges, which define a linkage pattern ABC with a linkage profile of 1 (occurrence of 1). In the meantime, the A and B events are both connected to drug 2 one time, therefore defining another linkage pattern AB with a linkage profile of 1. Similarity, in G^b2^, the linkage pattern AB has a linkage profile of 2 as it appears two times (drugs 1 and 2). The linkage profile of linkage pattern ABCDE is 1 (drug 3). Then, from the bipartite network, a projection to a monopartite network G^m^ is performed for the events. If two events share at least one common drug in the bipartite network, an association between these two events is generated. Finally, G^b1^ and G^b2^ were transformed into G^m1^, G^m2^, respectively. As observed, G^m1^ and G^m2^ are present as “cliques” meaning that all the events in G^m1^ and G^m2^ are fully interconnected. It is showed that the linkage pattern AB appears twice in G^b2^ while oit is overlapping as an edge AB in G^m2^, thus resulting in the information loss.

The relevance of the relations between nodes (events) might be underestimated when generating a monopartite network, and quantifying the importance of associations between pairs of events could be of interest for AOPs construction (Figure 2). To evaluate this issue in the developed AOP network, we applied the approach of loss of information as defined previously by Vogt and Mestres (30) and implemented two measures that are the “increase of uncertainty” and the “loss of coverage”.

### Increase in Uncertainty

The concept of “uncertainty” came from the Shannon entropy (31). It measures how many associations between drugs and events are spread over all possible connections in a bipartite network. In the bipartite network of the present study, the associations between two types of nodes (i.e., drugs and events) define a “linkage pattern.” After a projection to a monopartite network, only pairs of events having at least one common drug will be kept. Using the Shannon entropy theory, the distribution and the occurrence of each linkage pattern in the bipartite network can then be estimated in the projected monopartite network providing a measure of the increase in uncertainty (or loss of information) as presented in the equation below:


$$\begin{array}{l} {\text{H}}^{\text{before}}=-\overset{\left|{\text{L}}^{{\text{G}}^{\text{b}}}\right|}{\underset{\text{i}=1}{\sum }}\text{ p }({\text{I}}_{\text{i}})\;\cdot \;[\text{ln p }({\text{I}}_{\text{i}})],\;\;{\text{I}}_{\text{i}}\in {\text{L}}^{{\text{G}}^{\text{b}}}\\ {\text{H}}^{\text{after}}=-\overset{\left|\text{cliques}({\text{G}}^{\text{m}})\right|}{\underset{\text{i}=1}{\sum }}\text{p}({\text{C}}_{\text{i}})\;\cdot \;[\text{ln p }({\text{C}}_{\text{i}})],\\ \text{ΔH}={\text{H}}^{\text{after}}-{\text{H}}^{\text{before}} \end{array}$$

where H^before^ indicates the uncertainty of the linkage patterns of events in bipartite network (G^*b*^); L^G^b^^ represents the linkage patterns in the bipartite network including events; and p(I_i_) means the probability of each linkage pattern among all the linkage patterns in the bipartite network. After projection into the monopartite network G^m^, H^after^is used to estimate the uncertainty of G^m^. Since G^m^ is the undirected uncertain network, if a set of nodes are fully interconnected in a subnetwork of G^m^, this subnetwork is defined as “clique”. A clique in the uncertain network tends to be a deterministic subnetwork (32). Here, |cliques(G^m^)| indicates all the cliques in the monopartite network. p(C_i_) represents the probability of each clique in G^m^. ΔH describes quantitatively information in G^m^ compared with G^b^. The unit of increase in uncertainty ΔH (nats) is based on the natural logarithm. The lower the nats value, the lower amount of information is lost in the monopartite network.

Compared with the calculation of H^before^ for which all possible linkage patterns (L^G^b^^) in the bipartite network are considered, H^after^ focus on all possible cliques (|cliques(G^m^)|) in monopartite network. For a monopartite network with a huge number of events, it is difficult to count manually the number of cliques. Thus |cliques(G^m^)| can be represented as the number of subcliques for a maximal clique in G^m^. A “maximal clique” is a clique that cannot be extended by adding another node of the network. For example, in Figure 2, the cliques in G^m1^ are {A},{A,B},{A,C}, {A,B,C}, while the maximal clique is only {A,B,C}. The number of events included in a maximal clique is defined as the “size of a maximal clique.” Thus the size of maximal clique in G^m1^ is 3 (i.e., {A,B,C}). Then, for the calculation of subcliques, we applied the formulation: Number of subcliques for a maximal clique = 2^|c|^, where |c| is denoted as the size of a maximal clique (30). For example, with G^m1^, it suggests that there exists 2^|3|^ possible subcliques in the monopartite network. Although the calculation of the number of subcliques by these formulas can lead to bias compared with the reality (8 subcliques vs. 3 subcliques), it can still provide approximate values by calculating the maximal clique for the monopartite network of large size.

In our work, we calculated two kinds of uncertainty, H^before^, both in terms of global network uncertainty and nodes (events) uncertainty. ${\text{H}}_{\text{network}}^{\text{before}}$ is defined as the initial uncertainty of the global network. The calculation of ${\text{H}}_{\text{network}}^{\text{before}}$ takes into account all the linkage patterns in the bipartite network. Thus ${\text{H}}_{\text{Gb}1}^{\text{before}}=-\left[\text{ln}\left(\frac{1}{2}\right)\times \left(\frac{1}{2}\right)+\text{ln}\left(\frac{1}{2}\right)\times \left(\frac{1}{2}\right)\right]=0.69\text{ nats}$. At the difference, ${\text{H}}_{\text{nodes}}^{\text{before}}$ (the initial uncertainty in terms of nodes) only considers the linkage patterns related to a specific node (event) for the calculation. For example, to calculate ${\text{H}}_{\text{C}}^{\text{before}}$ for C in G^b1^, only linkage pattern related to the event “A,B,C” was considered in the calculation. Thus, ${\text{H}}_{\text{C}}^{\text{before}}=-\left[\text{ln}\left(\frac{1}{2}\right)\times \left(\frac{1}{2}\right)\right]=0.35\text{ nats}$. Similarly, after the projection to the monopartite network, ${\text{H}}_{\text{network}}^{\text{after}}$ will include all the cliques for the calculation i.e., ${\text{H}}_{\text{Gm}1}^{\text{after}}=-\left[\text{ln}\left(\frac{1}{8}\right)\times \left(\frac{1}{8}\right)\times 8\right]=2.08\text{ nats}$. For the nodes, ${\text{H}}_{\text{nodes}}^{\text{after}}$ considers the maximal clique contained in a specific node. So in theory, ${\text{H}}_{\text{C}}^{\text{after}}=-\left[\text{ln}\left(\frac{1}{8}\right)\times \left(\frac{1}{8}\right)\times 8\right]=2.08\text{ nats}$ for the event C in G^m1^. Overall, the resulting ΔH_network_ = 1.39 nats represents an increase in uncertainty for the global network during the transformation from G^b1^ to G^m1^. The H_nodes_ for C (ΔH_C_ = 1.73 nats) indicates that linkage patterns related to the C event has an increase in uncertainty of 1.73 nats after translating into cliques.

A high ΔH means that the monopartite network projection of the bipartite network has lost information during the transformation, suggesting an increase of uncertainty on the linkage pattern between events. Also, for the nodes, a high ΔH (like for C) depicts a low linkage of this event compared with the other events inside the clique. Therefore, these ΔH values can quantify the reliability of a connection between an event and other events depending on the drug–event information integrated into the original network.

### Loss of Coverage

In addition to the loss of information estimation, it is also possible to determine the degree of involvement of each edge during the projection, which indicates the original properties of the linkage pattern lost during monopartite network projection. This is the “loss of coverage,” and it is calculated using the formulations below:


$$\begin{array}{l} {\text{ COV}}_{\text{Edge}}=\frac{{\text{Σ}}^{\left|{\text{L}}_{\text{vw}}^{\text{b}}\right|}{\text{ n}}_{\text{i}}\;\cdot \;\frac{\text{2}}{{\text{I}}_{\text{i}}\text{V}}}{{\text{Σ}}^{\left|{\text{L}}_{\text{vw}}^{\text{b}}\right|}{\text{ n}}_{\text{i}}},{\text{with COV}}_{\text{Edge}}\in (\text{0},\text{1})\\ \neg {\text{COV}}_{\text{Edge}}=\text{1}-{\text{COV}}_{\text{Edge}},\text{with }\neg {\text{COV}}_{\text{Edge}}\in (\text{0},\text{1}) \end{array}$$

The term COV_Edge_ is the coverage for each edge between events v and w in G^m^. ${\text{L}}_{\text{vw}}^{\text{b}}$ is the amount of linkage patterns, I_i_V contains both events v,w in the bipartite network. Here, n_i_ is the occurrence of I_i_V. ¬COV_Edge_ indicate the loss of coverage for the edge between event v and w in G^m^. A high ¬COV_Edge_ (maximum is 1) indicates that the edge between the two events presented in the monopartite network cover few commun information (for example few number of drugs) and a ¬COV_Edge_ close to 0, which means that many drugs are linked to two events in the bipartite network. For example, to calculate COV_Edge(A,B)_ for G^m1^, the linkage patterns containing A and B are “AB” and “ABC”. Both linkage patterns appear once in the G^b1^. So, for $\text{CO}{\text{V}}_{\text{Edge}\left(\text{A},\text{B}\right)}=\frac{\left[1\times \left(\frac{2}{2}\right)+1\times \left(\frac{2}{3}\right)\right]}{1\;+\;1}=0.83\;\neg \text{CO}{\text{V}}_{\text{Edge}\left(\text{AB}\right)}=0.17$. Doing a similar analysis between B and C, we obtained a ¬COV_Edge(BC)_ = (0.33) in G^m1^. So, the coverage is more important between events A and B than between B and C.

Finally, we computed the loss of coverage for nodes ¬COV_Node_ in the monopartite projection. It considers the average of all the edges in G^m1^ that are linked to a node. For example, the event C is linked to the event B and A. Therefore, the loss of coverage for the event C is $\neg \text{CO}{\text{V}}_{\text{C}}=\frac{\;\neg \text{CO}{\text{V}}_{\text{Edge}\left(\text{B},\text{C}\right)\;}+\;\neg \text{CO}{\text{V}}_{\text{Edge}\left(\text{A},\text{C}\right)}}{2}$ = 0.33. This value can then be compared with the loss of coverage for all other events within the aim to assess the coverage associated with each event in the monopartite network.

### Visualization and Analysis of the Loss Information of the Developed Network

In both the methods, an increase in uncertainty and loss of coverage were implemented under python 3.7 and R, using the R package Reticule as an interface provider between R and Python

(https://cran.r-project.org/web/packages/reticulate/reticulate.pdf) (R version 3.5.3). All implemented methods and applications in this work are available on GitHub (https://github.com/QierWU/drug-AOP). Results were visualized and analyzed in Cytoscape V3.7.2.

## Results

### Drug–Event Data

Drug–event associations were compiled from two complementary data sources and merged to obtain unique relationships. From the CompTox database, we were able to collect 248 drugs associated with 23 unique MIEs (as of March 2021) (Supplementary Table S1). From the AOP-wiki database, we extracted 117 unique drugs having at least one corresponding event (as of August 2020) (Supplementary Table S1). However, 46 among these 117 drugs were only connected to an AOP, with no information concerning the event linkage (MIE, KE, AO). For example, meclofenamic acid was associated with the AOP 152 'Interference with thyroid serum binding protein transthyretin and subsequent adverse human neurodevelopmental toxicity,” but no KE was specified. Therefore, to develop a more complete network, we manually explored the literature using the PubMed database to enrich the connection between the drugs and the 11 events known to be involved in the AOP 152 (Supplementary Table S2). For meclofenamic acid, 1,158 publications were identifided. Then, we searched among these abstracts, those mentioning KE of interest. As an example, the MIE 957 “Binding, Transthyretin in serum,” which is know to be linked to the AOP 152 (33) was retrieved in 5 out of the 1,158 abstracts. With this curation, we were able to decipher the linkage between 46 drugs and 18 events *via* 41 publications. As a result, drugs were connected to 89 unique events (52 MIEs, 31KEs, 6 AOs).

In addition, to enrich the drug–event associations network, we used KERs information that link event pairs from the AOP–wiki database (as of September 2020) (Supplementary Table S3). For example, the MIE 718 “Binding, Tubulin” and the AO 728 “Increase, Aneuploid offspring” were present in the developed bipartite network (Supplementary Table S1). From the AOP–wiki, both are also part of the AOP 106 “Chemical binding to tubulin in oocytes leading to aneuploid offspring” (https://aopwiki.org/aops/106, as of September 2021). Therefore, events involved in AOP106 were added into our bipartite network. This step allowed to integrate information related to 13 KEs. Overall, all obtained information were merged to keep only unique information to build the bipartite network, which consists of 321 drugs, 66 MIE, 44 KE, and 6 AO (Supplementary Table S4).

### Development of the Drug–Event Bipartite Network

**Figure 3:**
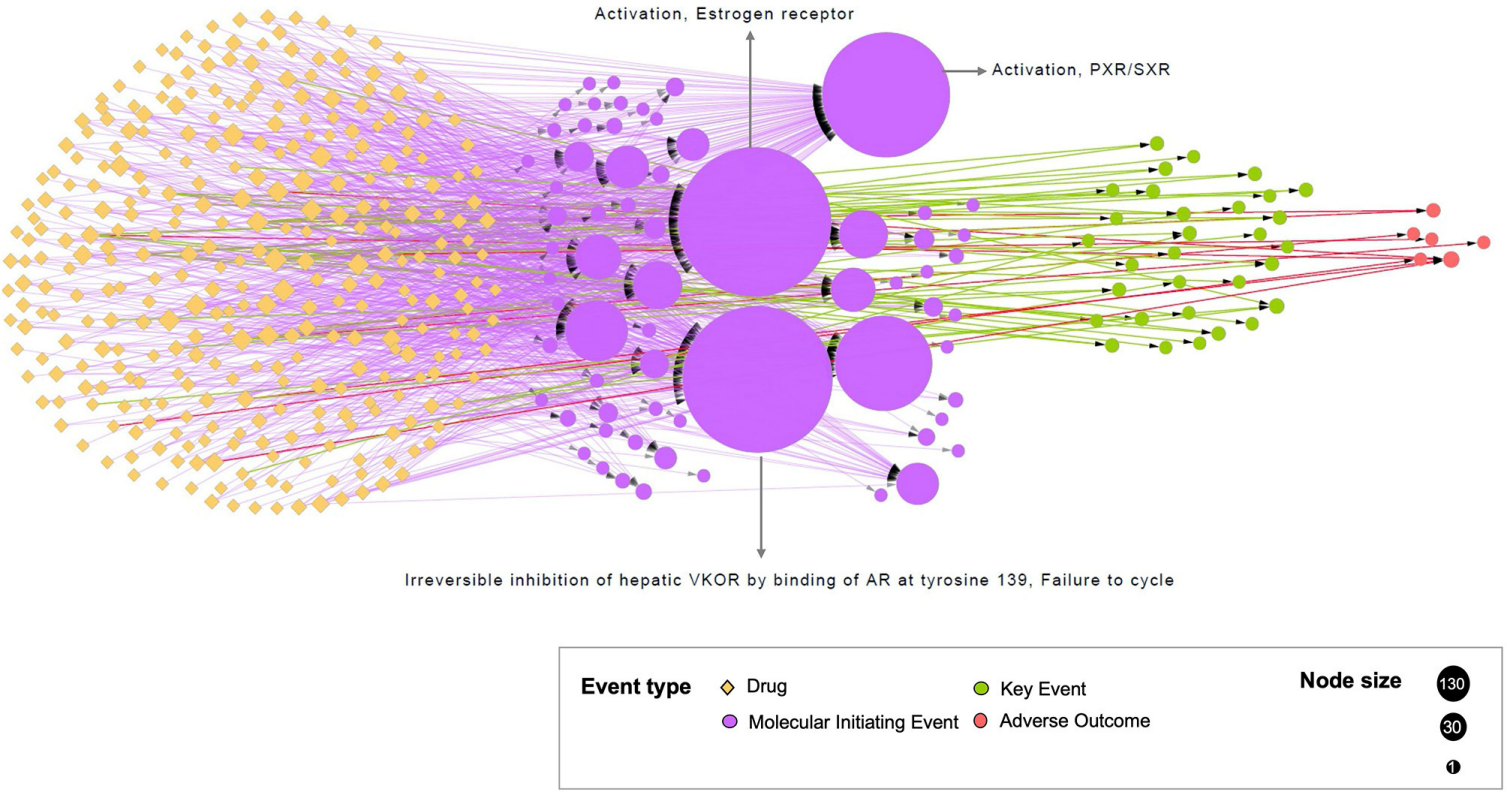
Representation of the bipartite network. The network illustrates the drug–event associations data compiled from the CompTox and the AOP-wiki databases. Each circle node represents one event, colored by the event type to which it belongs (MIE, KE or AO). Each yellow diamond node represents one drug. The size of the event node corresponds to the number of drugs known to be linked to event(s) (from 1 to 134). The size of the drug node corresponds to the number of events associated with this drug (from 1 to 12). The added KEs, using the KERs information from the AOP-wiki database, are in directed green dash lines. Directed solid lines indicate direct drug–event associations extracted during te compilation phase. These edges are colored by the event type to which drugs are connected i.e., drug-MIE in purple, drug-KE in green, and drug-AO in red.

All the 321 drugs and their direct associations to events were connected through 981 edges in the bipartite network (Figure 3). All the events were classified into three groups based on their different event types (MIE, KE, and AO). Interestingly, many drugs were linked to at least one nuclear receptor: 134 drugs were connected to the MIE 1181 “Activation, Estrogen receptor,” 131 drugs were connected to the MIE 1134 “Irreversible inhibition of hepatic VKOR by binding of AR at tyrosine 139, Failure to cycle vitamin K epoxide to vitamin K to form vitamin K hydroquinone,” and 110 drugs were connected to the MIE 245 “Activation, PXR/SXR.” Among the most linked drugs, propylthiouracil, used to treat hyperthyroidism was connected to the maximum number of events (12 events). The antifungal agent, clotrimazole, was connected to 11 events, and the antiinflammatory drug, betamethasone, was connected to 9 events. It was also observed that 134 drugs were only associated with a unique event (e.g., Sertraline, Topiramate). Globally, the drug–event network emphasized that most of the studies focused on interactions between drugs and adverse protein targets.

### Monopartite Network of Events in the Drug Space

**Figure 4:**
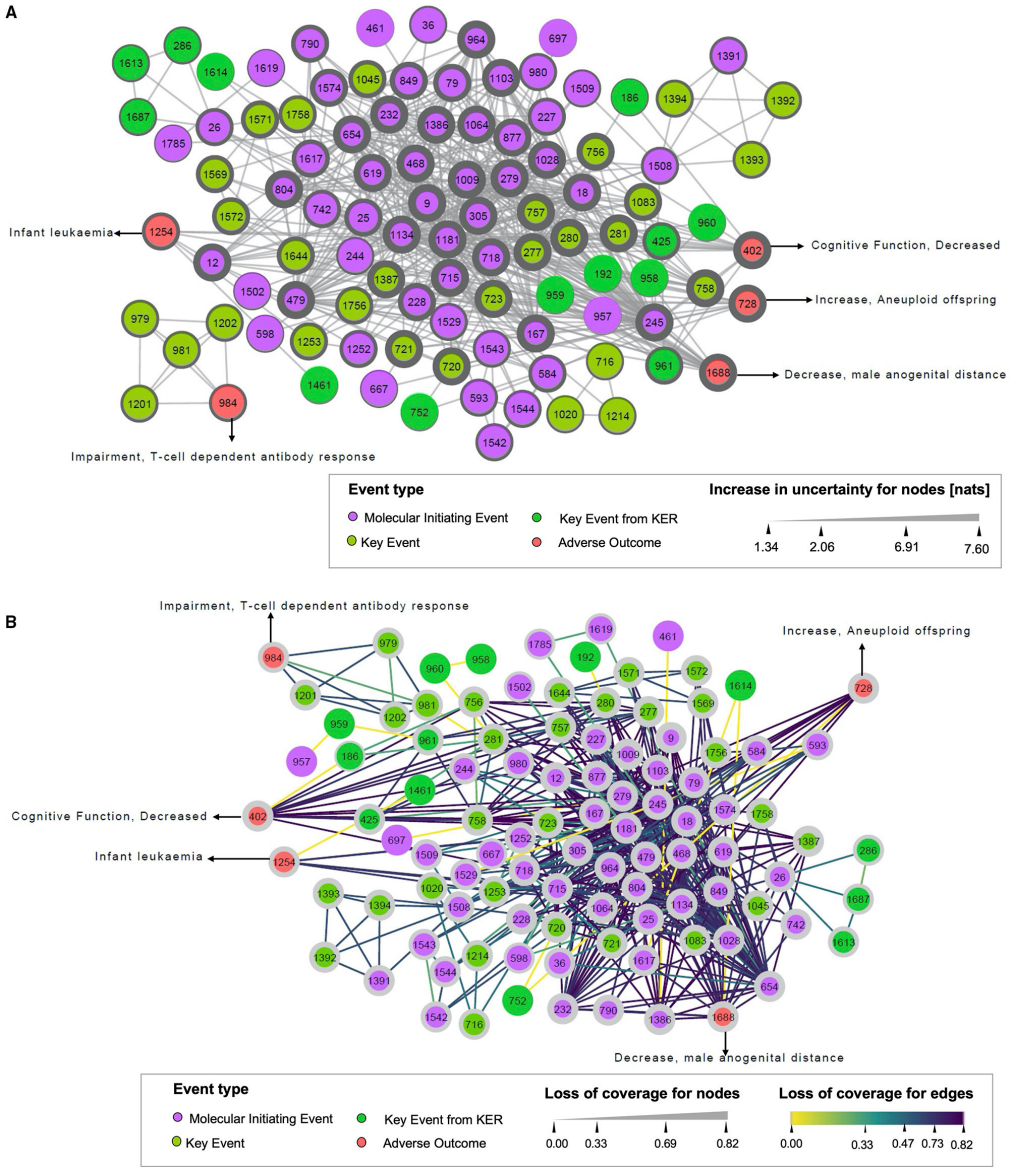
Uncertainty increase **(A)** and loss of coverage **(B)** of the monopartite network. (A) Illustrates the increase in uncertainty, and **(B)** represents the loss of coverage, both for the monopartite network of events. Each circle node represents one event, colored by the event type to which it belongs (MIE, KE, AO). Solid edges indicate event–event associations. In **(A)**, the width of nodes increase according to the value of the increase in uncertainty. In **(B)**, the width of nodes is raised with the value of loss of coverage. The dark color of the edges shows the highest value of loss of coverage.

The developed monopartite network projection from the drug-AOP bipartite network is represented in Figure 4. It was built based on the assumption that if two events in the drug-event network shared at least one common drug or event, a link between these two events can be depicted in the monopartite network. Fourteen events (1AO, 2KEs, 11MIEs) were linked to a unique drug and were not included in the monopartite projection (as these KEs were not interacting with other KEs). At the end,102 events were connected through 515 edges.

Considering the increase in uncertainty (Figure 4A), nodes with thicker widths indicated more information loss. The MIE 9 “Activation, 5HT2c” and MIE 1009 “Inhibition, Deiodinase 1” had the highest increase in uncertainty (7.60 nats), reflecting that these two events have a linkage to a unique (or few) drug(s), but this (or these) drug(s) are connected to many events. The high value of an increase in uncertainty is related to the maximum size of linkage pattern associated with the MIE 9 or MIE 1009 in the bipartite network. In fact, the MIE 9 was only connected to the drug “clove leaf oil” in the bipartite network. Moreover, this drug was also linked to 10 other MIEs. It suggested that 2^10^possible linkage patterns relevant to the MIE 9 could exist in the bipartite network. Obviously, the number of linkage patterns related to the MIE 9 projected in the monopartite network is less. Thus, the uncertainty for the linkage patterns relevant to the MIE 9 increased largely after monopartite network projection. On the contrary, it is observed that the KE 1614 “Decrease, AR activation” had the lowest increase in uncertainty (1.34 nats), meaning that this KE had a small size of linkage patterns in the bipartite network, thus resulting in low uncertainty in the monopartite network. Regarding loss of coverage (Figure 4B), the nodes with thicker width and purple edges had high information loss. The value of ¬COV_Edge_ gives the coverage loss between two nodes in a monopartite network, whereas the value of ¬COV_Node_ defines the mean of ¬COV_Edge_ associated with a node. For example, the edge between the MIE 9 and the MIE 18 “Activation, AhR” has a value of ¬COV_Edge(MIE 9, MIE 18)_ = 0.82. Such ¬COV_Edge_ can be considered as high because it is close to 1. On the opposite, the MIE 167 “Activation, LXR” and the MIE 468 “Inhibition, PPAR alpha” shared 10 drugs. The loss of coverage will be lower with ¬COV_Edge (MIE167, KE468)_ = 0.75, leading to a higher confidence about the relationship between these two events. Similarly, the drug propylthiouracil, which targets the MIE 1009, is also linked to 10 other events, among them the MIE 18 “Activation, AhR” and the MIE 245 “Activation, PXR/SXR.” These two MIEs have 19 drugs in common, resulting in a weak loss of coverage (¬COV_Edge(MIE18, MIE245)_ = 0.61) between these two events, and a higher chance that a drug impacting one of this event might has also an effect on the second event (Supplementary Figure S1). Similarly, for the edge between MIE 1181 “Activation, Estrogen receptor” and MIE 1543 “Mitochondrial Complex IV inhibition,” the ¬COV_Edge(MIE 1181, MIE 1543)_ = 0.33, meaning a limited loss of coverage.

Additionally, a loss of information can be computed on each node (¬COV_Node_). ¬COV_Node_ is the mean of ¬COV_Edge_ associated with a node. For example, the MIE 9 and the MIE 18 are connected with a ¬COV_Edge(MIE 9, MIE 18)_ = 0.82, resulting at the node level to ¬COV_MIE9_ = 0.82 and also ¬COV_MIE18_ = 0.72. This suggests that the putative associations related to the MIE 9 and the MIE 18 in the monopartite network simplified the real situation of connections in the bipartite network.

Finally, we noticed a loss of coverage equal to zero between the two events, the MIE 1529 “Blockade, L-Type Calcium Channels” and the MIE 593 “Inhibition, Ether-a-go-go (ERG) voltage-gated potassium channel.” It means there is no loss of information during the projection. This suggests a very strong relationship between these two events. However, in very specific cases, the loss of coverage might be equal to zero when a linkage pattern has a size of two, and the linkage profile is more than once. In this case, loss of coverage does not reflect the reality. For example, the KE 1614 “Decrease, AR activation” and AO 1688 “decrease, male anogenital distance” shared with two common KEs that are very similar (KE 1687 “decrease, transcription of genes by AR” and KE 286 “Altered, Transcription of genes by AR”). The size of the linkage pattern as well as the linkage profile is two. As these associations are merged as one unique edge in the monopartite network, the value of loss of coverage at the edge is 0. To better illustrate the use of this events network, a case study related to infertility is presented in Supplementary Material.

In a global overview, the information loss for the full monopartite network and each type of event (MIE, KE, and AO) are summarized in Table 1. The initial uncertainty (${\text{H}}_{\text{network}}^{\text{before}}$) for the network is 5.00 nats, and for the nodes ( is 0.05 nats. Among the subnetworks of ${\text{H}}_{\text{nodes}}^{\text{before}}$, the MIE nodes showed the largest initial uncertainty value (0.08 nats), whereas the KE and AO showed the lowest value (0.03 nats). This indicated that the linkage patterns related to nodes in the MIE subgroup had a higher initial uncertainty compared with the AO and KE subgroups. All the nats values for the 3 subgroups are close to 0 and so the initial uncertainty at node level is low. After the monopartite projection, the uncertainty at the global network (${\text{H}}_{\text{network}}^{\text{after}}$) and nodes (${\text{H}}_{\text{nodes}}^{\text{after}}$) is 11.28 and 5.06 nats, respectively. This means that the uncertainty increased by 6.28 nats (ΔH_network_) for the monopartite network. A 5.01 nats increase is observed in terms of nodes (ΔH_nodes_). For subnetworks, the increase in uncertainty (ΔH_nodes_) is of 5.79 nats for the AO and 4.15 nats for the KE. This reflects that the projection procedure has more impact on the AO than the KE.

**Table 1:** Information loss for the monopartite network.

	** ${{\text{H}}^{1}}_{\text{n}\text{etwor}\text{k}}^{\text{b}\text{efor}\text{e}}$ **	** ${{\text{H}}^{2}}_{\text{n}\text{ode}\text{s}}^{\text{b}\text{efor}\text{e}}$ **	** ${{\text{H}}^{3}}_{\text{n}\text{etwor}\text{k}}^{\text{a}\text{fte}\text{r}}$ **	** ${{\text{H}}^{4}}_{\text{n}\text{ode}\text{s}}^{\text{a}\text{fte}\text{r}}$ **	** ${{\text{Δ}\text{H}}^{5}}_{\text{n}\text{etwor}\text{k}}$ **	** ${{\text{Δ}\text{H}}^{6}}_{\text{n}\text{ode}\text{s}}$ **	** ${\neg {\text{C}\text{O}\text{V}}^{7}}_{\text{E}\text{dg}\text{e}}$ **	** ${\neg {\text{C}\text{O}\text{V}}^{8}}_{\text{N}\text{od}\text{e}}$ **
Network	5.00	0.05	11.28	5.06	6.28	5.01	0.67	0.58
AO	–	0.03	–	5.82	–	5.79	0.68	0.64
KE	–	0.03	–	4.18	–	4.15	0.62	0.50
MIE	–	0.08	–	5.19	–	5.11	0.68	0.59

*Adverse outcome (AO), key event (KE), and molecular initiating event (MIE)*.

The average loss of coverage for edges (¬COV_edge_) and nodes (¬COV_node_) after projection in the monopartite network are 0.67 and 0.58 respectively. Higher values of ¬COV_edge_ and ¬COV_node_ are observed for AO compared with KE and MIE, revealing a higher loss of information for AO.

${{\text{H}}^{1}}_{\text{network}}^{\text{before}}$: initial uncertainty in terms of the network, ${{\text{H}}^{2}}_{\text{nodes}}^{\text{before}}$: initial uncertainty in terms of nodes. The value of each subgroup was an average of H^before^ for each node in the given subgroup; ${{\text{H}}^{3}}_{\text{network}}^{\text{after}}$: uncertainty after projection for the network; ${{\text{H}}^{4}}_{\text{nodes}}^{\text{after}}$: uncertainty after projection for nodes. Value of each subgroup was average of H^after^ for each node in the given subgroup; ${\text{Δ}{\text{H}}^{5}}_{\text{network}}$: increase in uncertainty for the network; ${\text{Δ}{\text{H}}^{6}}_{\text{nodes}}$: increase in uncertainty for nodes; ${\neg \text{CO}{\text{V}}^{7}}_{\text{Edge}}$: loss of coverage at the level of edge. For the value of each subgroup, considering all the edges that link nodes belonged to the target subgroup, the average of loss of coverage for each edge was calculated; ${\neg \text{CO}{\text{V}}^{8}}_{\text{Node}}$: loss of coverage for nodes. The value of each subgroup was the average of loss of coverage for each node in the given subgroup.

## Discussion

Assessment of the chemical toxicity at an early stage remains a tremendous challenge for the pharmaceutical industries and the regulatory agencies. To have a better picture of the effects of the chemical exposome on human health, new and innovative concepts and methods are needed. Even if various systems toxicological models have been developed to decipher chemical toxicity in humans, there is still a need to increase our understanding (34, 35).

In this study, we proposed a network-based approach that supports the concept of integrated approaches to testing and assessment (IATA) proposed by the OECD (36). Based on our method, we explored the potential biological mechanisms of drugs that lead to adverse effects by analyzing AON that result from AOPs. Network-based modeling has the advantage of being a chemical structural information free and can be used to establish toxicological profiling for substances. The developed process is part of a new approach methodology (NAM), which supports the use of alternative methods to animal testing for the identification of biological perturbation from chemical exposure (37).

Here, we first developed a bipartite network model to capture the behaviors of drug–event associations described by the AOP concept based on the assumption that the real-world complex networks have bipartite structures (38, 39). The bipartite network was then projected into a monopartite network to analyze the relationship between the two events. However, this translation is often accompanied by a loss of information (40), and we decided to quantify the loss of information and the coverage obtained within this events network.

The generated events network was evaluated using a method that quantifies the reduction with probability in two ways: “increase of uncertainty” and “loss of coverage.” Results indicated that the level of information loss during monopartite projection mainly depends on the number of events involved in a linkage pattern (also called the size of linkage pattern), which is illustrated by a case study with the health effect “infertility” (Supplementary Material). Therefore, such methods allow to identify uncertainties and data gaps between the different events involved in an AON, and also to quantify the confidence of a relation between the two events.

Although this method is able to illustrate links between drugs and AOPs, one limitation of the developed network is that it relies on existing information. Some causal linkages may have been overlooked or disregarded because of missing or incomplete information. Morever such modeling study should take into consideration the so-called “Matthew effect,” that reflects the difference, in terms of available knowledge (database, literature), between the very well investigated drugs and the ones less studied (41). Therefore, an extension of such model could be done by integrating more data, including quantitative information, and to fully evaluate the toxicological effects of drugs. One way could be to adapt existing tools such as AOP-helpFinder, which uses artificial intelligence and text mining to automatically screen the literature, to compile more data (42, 43). For example, this tool was successfully applied to a set of pesticides by automatically identifying among the 32 million abstracts present in the PubMed database, links between pesticides and events (44). Another way would be to gather information from publicly available databases, which should become more accessible within the next few years following the interoperability of the data based on the goals established by Findable, Accessible, Interoperable, and Reusable (FAIR) data principles (45). There is a need to implement standardization of data to facilitate the use of multiple data sources, as a lack of interporability between all existing databases increases the difficulties for developping integrative models.

Other data types could also be considered to have a more complete model as possible, reflecting therefore the biological “true.” As an example, environmental chemicals, pharmacogenomics, or drug–drug interactions could also be taken into consideration.

All these data should allow the development of predictive models and emerging tools. Nevertheless, the challenge will be on how to integrate all these heterogeneous data (from various species, different technologies…) in a common platform (46). Finally, our study relies on the available names of the KEs extracted from existing databases, and some KEs may have a similar biological meaning (e.g., in AOP-wiki, KE1608 “Increase, Oxidative DNA damage” and KE1634 “Increase, Oxidative damage to DNA”). Therefore, there is a need to harmonize the KE nomenclature to develop the best predictive models possible.

## Conclusions

A network-based model was developed to explore in a systematic manner how drugs may be linked to biologic events (MIE, KE, and AO), and to quantify the uncertainty of a relation between events within an AOP. This method could be of interest as a weight of evidence (WoE) evaluation and could be complementary to the systematic WoE analysis provided so far (47). Overall our analysis highlights that such systems' toxicology science can help to improve the knowledge of interactions between drugs and biological systems, which can be of great interest for animal-free next generation risk assessment (NGRA).

## Data Availability Statement

The original contributions presented in the study are included in the article/Supplementary Material, further inquiries can be directed to the corresponding author/s.

## Author Contributions

QW: data curation, formal analysis, methodology, result analysis, and drafted the manuscript. YB: software development. OT: design the study, result analysis, and revised the paper. KA: design the study, result analysis, revised the paper, and supervision. All authors contributed to the article and approved the submitted version.

## Funding

This work was supported by the Université de Paris and the French National Institute of Health and Medical Research (Inserm).

## Conflict of Interest

The authors declare that the research was conducted in the absence of any commercial or financial relationships that could be construed as a potential conflict of interest.

## Publisher's Note

All claims expressed in this article are solely those of the authors and do not necessarily represent those of their affiliated organizations, or those of the publisher, the editors and the reviewers. Any product that may be evaluated in this article, or claim that may be made by its manufacturer, is not guaranteed or endorsed by the publisher.
