# Supplementary material for: Capturing a Comprehensive Picture of Biological Events From Adverse Outcome Pathways in the Drug Exposome
Source: Front Public Health. 2021 Dec 17;9:763962. doi: 10.3389/fpubh.2021.763962 (PMC8718398; doi:10.3389/fpubh.2021.763962)
Supplement: Supplementary file 1 [file Data_Sheet_1.docx]

**Supplementary Material**

**Capturing a comprehensive picture of biological events from Adverse Outcome Pathways in the drug exposome**

Qier Wu^1^, Youcef Bagdad^1^, Olivier Taboureau^2^, Karine Audouze^1^

**Affiliations :**

^1^ Université de Paris, T3S, Inserm UMR S-1124, F-75006 Paris, France

^2^ Université de Paris, Inserm U1133, CNRS UMR 8251, F-75013 Paris, France

**Supplementary information (SI) for drug-AOP.**

Figure S1: Illustration of coverage for edges between MIE 18 ‘Activation, AhR’, MIE 245 ‘Activation, PXR/SXR$' \left( {\neg COV}_{\mathrm{Edge}\left( MIE18,MIE245 \right)} \right)$

Table S1: Source information from AOP-wiki and CompTox (Supplementary tables: sheet 1)

Table S2: Drug-AOP events literature support. (Supplementary tables: sheet2)

Table S3: Source information of Key Event Relationship (KER). (Supplementary tables: sheet 3)

Table S4: Source information for the bipartite network 321 drugs-116 events. (Supplementary tables: sheet 4)

Table S5: Increase in uncertainty for nodes. (Supplementary tables: sheet 5)

Table S6: Loss of coverage for edges. (Supplementary tables: sheet 6)

Table S7: Loss of coverage for nodes. (Supplementary tables: sheet 7)

**Supplementary information (SI) for health effect infertility.**

Figure S2: Illustration of coverage for edges between KE 1614 ‘Decrease, AR activation’ and AO 1688 ‘decrease, male anogenital distance’$( {\neg COV}_{\mathrm{Edge}\left( KE1614,AO1688 \right)})$

Figure S3: Drug-event bipartite network for male anogenital distance.

Figure S4: Monopartite event projection in increase in uncertainty and loss of coverage for male anogenital distance.

Table S8: Increase in uncertainty for nodes for male anogenital distance. (Supplementary tables: sheet 8)

Table S9: Loss of coverage for nodes for male anogenital distance. (Supplementary tables: sheet 9)

Table S10: Loss of coverage for edges for male anogenital distance. (Supplementary tables: sheet 10)

Table S11: Information loss for male anogenital distance. (Supplementary tables: sheet 11)

**Supplementary information (SI) for drug-AOP.**


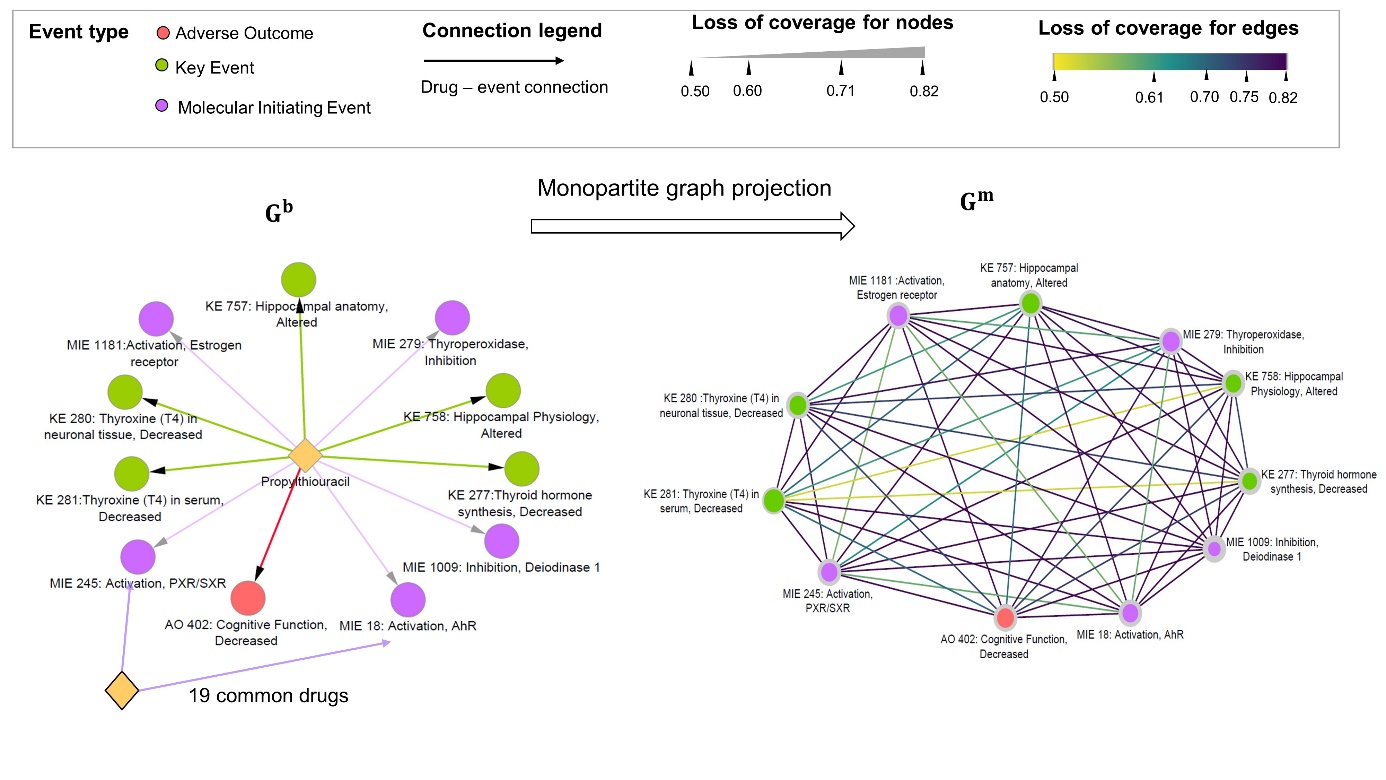


**Figure S1: Illustration of coverage for edges between MIE 18 ‘Activation, AhR’, MIE 245 ‘Activation, PXR/SXR**$\boldsymbol{'}\mathbf{(} {\neg COV}_{\mathrm{Edge}\left( MIE18,MIE245 \right)})$**.** On the left, G^b^ represents the bipartite network containing both MIE 18 and MIE 245. It is observed that both MIEs shared other 19 common drugs besides drug Propylthiouracil. Directed solid lines with arrows indicate known drug-AOP event associations. Each circle node represents one event, colored by the event type to which it belongs (MIE in purple, KE in green or AO in red). Each diamond node in yellow represents one drug. The figure on the right depicts the monopartite network G^m^ projected from G^b^. These associations are colored by the event type to which drugs are connected. Solid edges indicated event-event associations. The loss of coverage is represented with a grey color surrounding each node. The width of the grey color is related to the value of the loss of coverage. Dark color in edge accompanied with high value of loss of coverage at edge level.

**Table S1: Source information from AOP-wiki and CompTox** (See Supplementary tables: sheet 1)

**Table S2: Drug-AOP events literature support.** (See Supplementary tables: sheet2)

**Table S3: Source information of Key Event Relationship (KER).** (See Supplementary tables: sheet 3)

**Table S4: Source information for the bipartite network 321 drugs-116 events.** (See Supplementary tables: sheet 4)

**Table S5: Increase in uncertainty for nodes.** (See Supplementary tables: sheet 5)

**Table S6: Loss of coverage for edges.** (See Supplementary tables: sheet 6)

**Table S7: Loss of coverage for nodes.** (See Supplementary tables: sheet 7)

**SI for health effect infertility:**

In order to better illustrate the developed model, we performed a case study related to infertility. In clinical definition, anogenital distance (AGD) refer to the distance between anus and genitalia (1). AGD was associated with semen quality and considered as a potential biomarker to assess the reproductive toxicity of drugs (2–4). Several studies demonstrated that in utero exposure to drugs may impact the reproductive systems, resulting in a short anogenital distance in male(1,3,5). Short AGD in male is associated to testicular dysfunction(5–7). From the developed drug-AOP bipartite network, only the associations related to AO 1688 were selected to construct a specific bipartite sub-network (**Figure S2**). Loss of information (**Figure S3**) shows that the MIE 26 ‘Antagonism, Androgen receptor’ corresponds to a highest increase in uncertainty at node level (2.55 nats) wherevers KE 1614 “Decrease, AR activation” corresponds to the lowest increase in uncertainty at node level (1.02 nats) **(Figure S3A, Table S8 (sheet 8))**, meaning that MIE 26 had larger size of linkage patterns compare to KE 1614 in bipartite network, thus resulting in high uncertainty to MIE 26 and low uncertainty to KE 1614 in the monopartite network. The analysis also indicates that four events (MIE 26 ‘Antagonism, androgen receptor’, KE 1613 ‘Decrease, DHT level’, KE 286 ‘Altered, transcription of genes by AR’, KE 1687 ‘Decrease, transcription of genes by AR’) had a high${\neg COV}_{\mathrm{Edge}}$, and also a high${\neg COV}_{\mathrm{Node}}$ for these 4 events, meaning that the putative associations related to the these four events in monopartite network simplified the real situation of connections in bipartite network. **(Figure S3B, Table S9, Table S10 (sheet 9,10)**).

Information loss for infertility is summarized in **Table S11 (sheet 11)**. At the global network level, the global values of increase in uncertainty for infertility and nodes were 2.53 nats, 1.89 nats respectively. Loss of coverage related to the infertility network were 0.34 and 0.26 at the edge level and node level, respectively. For the subgroup nodes, it is noted that KE corresponds to largest increase in uncertainty at nodes level (2.11 nats). This subgroup nodes also have high loss of coverage et level of edge (0.37) and nodes (0.33). It is noted that no loss of coverage of AO 1688 was observed during monopartite projection. It means that the edges related to AO 1688 in monopartite network are originally presented as linkage patterns in bipartite network.


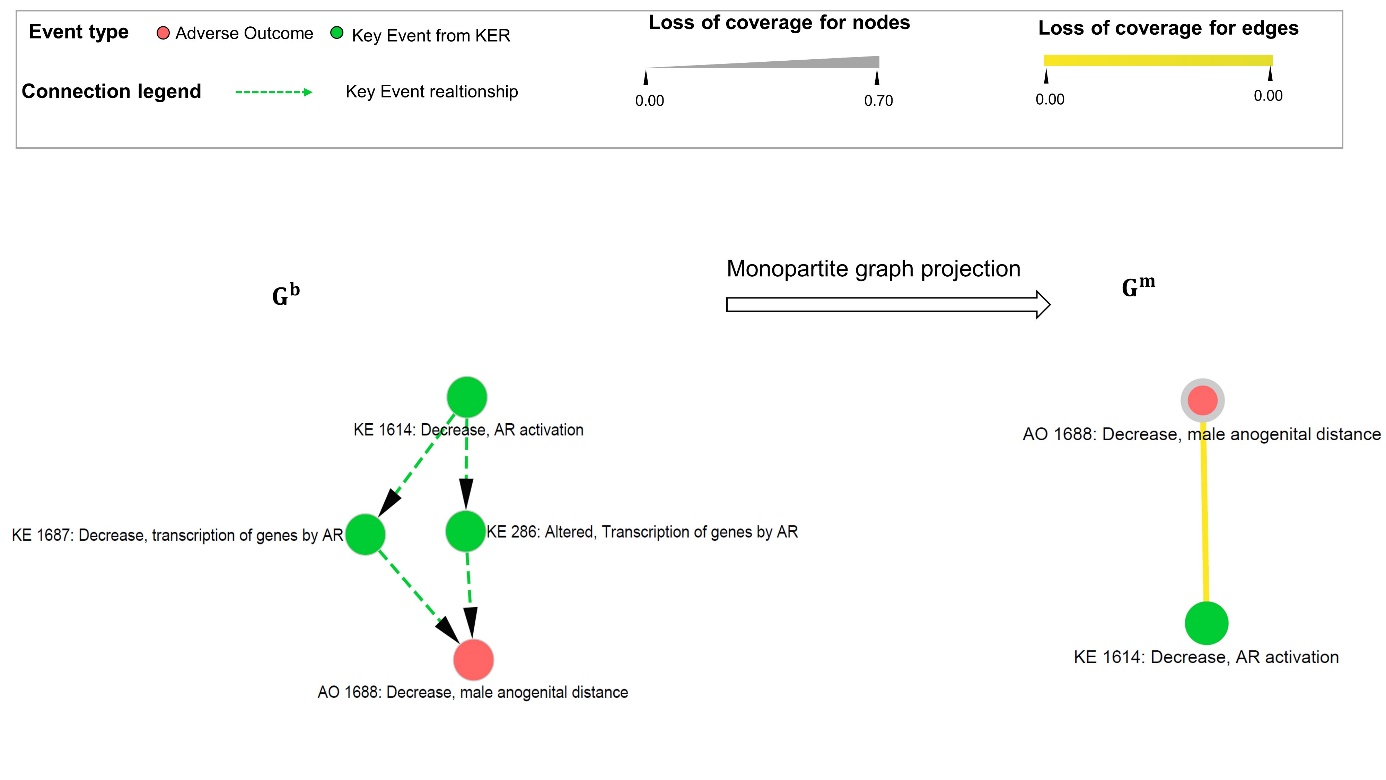


**Figure S2: Illustration of coverage for edges between KE 1614 “Decrease, AR activation” and AO 1688 “decrease, male anogenital distance”**$\mathbf{(} {\neg COV}_{\mathrm{Edge}\left( KE1614,AO1688 \right)})$**.** On the left, G^b^ represents the bipartite network containing both KE 1614 and AO 1688. They shared two common KEs that came from KER information. On the right, the monopartite network G^m^ is the one projected from G^b^. Each circle node represents one event, colored by the event type to which it belongs (KE in green or AO in red). In left G^b^, green directed dash lines indicate key events relationships (KER). In right G^m^, solid edges indicated event-event associations. The width of nodes colored in grey, were raised with value of loss of coverage (i.e, ${\neg COV}_{AO1688}$=0.70, ${\neg COV}_{KE1614}$=0.00).

**
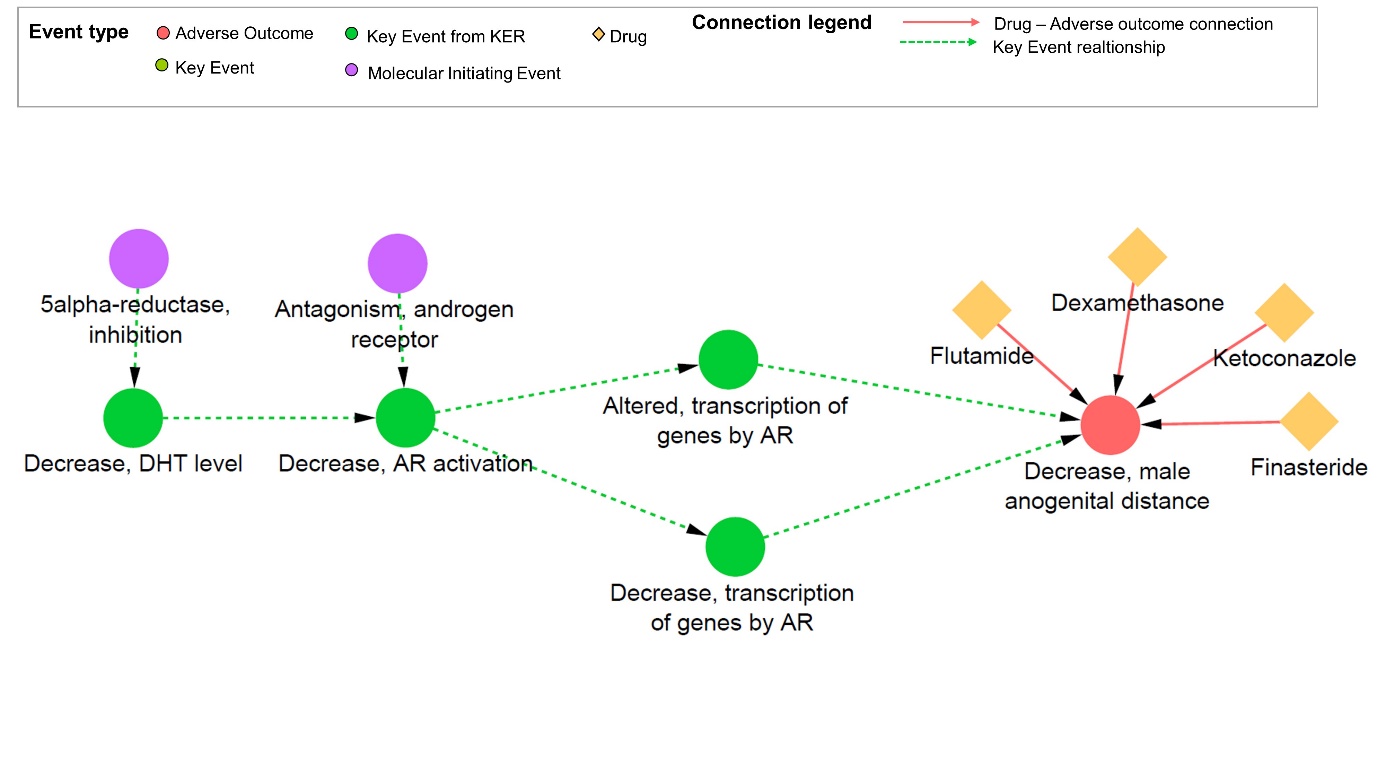
**

**Figure S3: Drug-event bipartite network for male anogenital distance.** Each circle node represents one event, colored by the event type to which it belongs (MIE, KE or AO). Each diamond node represents one drug. The KER are in directed green dash lines. Directed red solid lines indicate known drug-AO event associations.


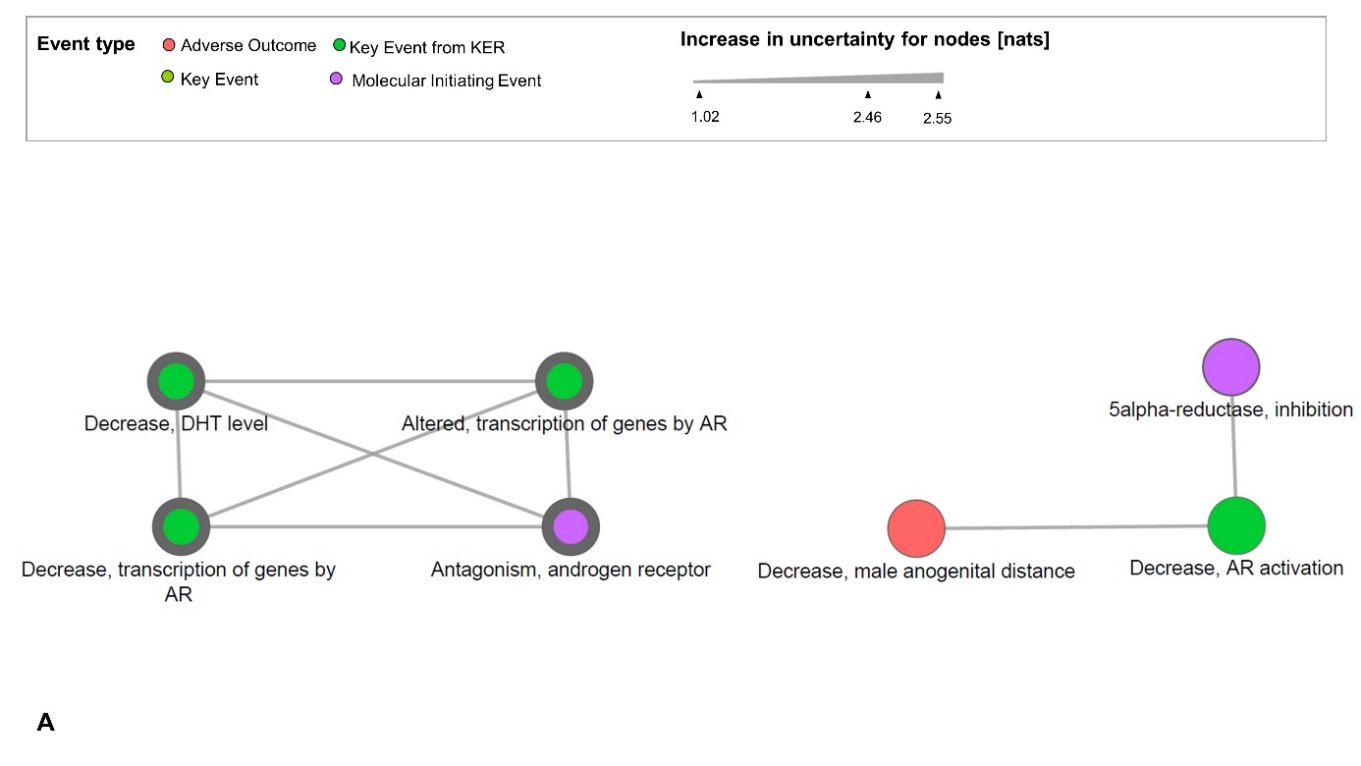

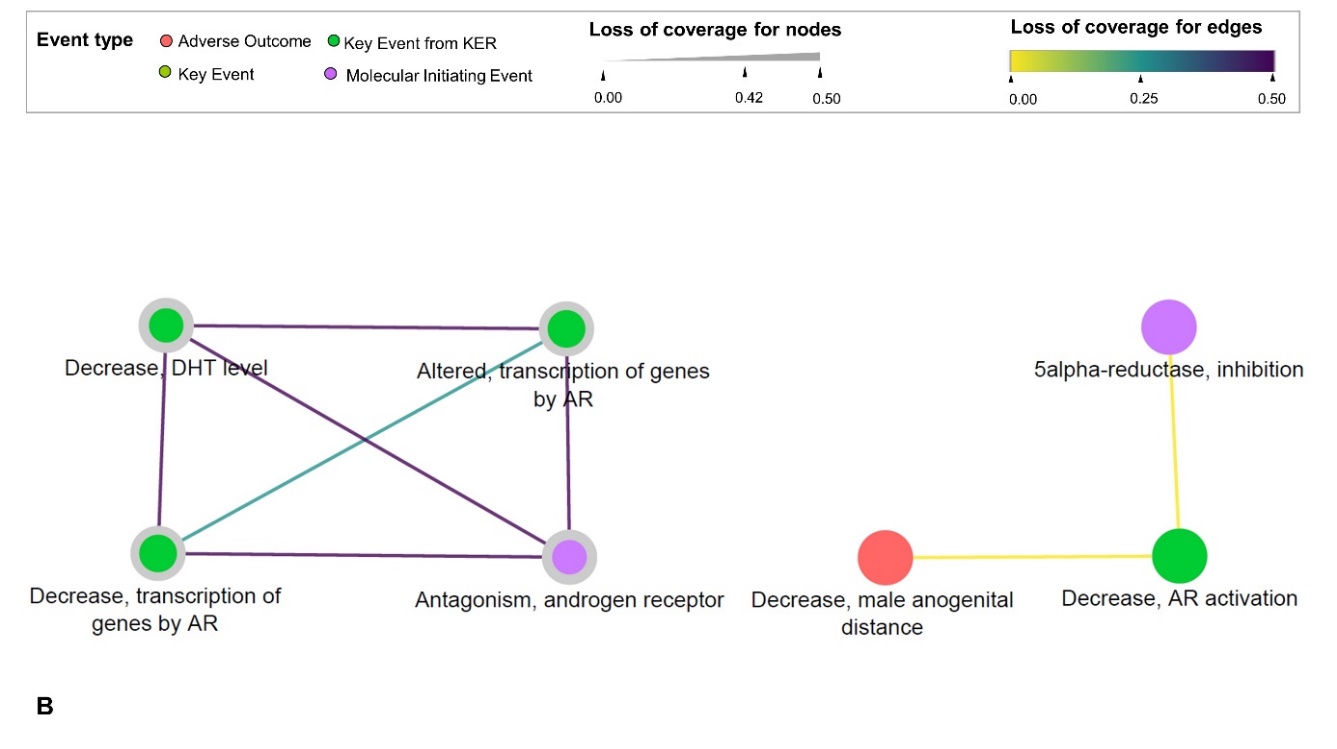


**Figure S4: (A) Monopartite event projection in increase in uncertainty and (B) loss of coverage for male anogenital distance.** Each circle node represents one event, colored by the event type to which it belongs (MIE in purple, KE in green and AO in red). Solid edges indicated event-event association. Figure S4A, illustrates the increase in uncertainty at the node level. The nats values are associated to the grey color surrounding the sphere. The larger is the grey area, the higher is the nats value. The Figure S4B represents the loss of coverage. The darker is the edge between 2 nodes, the higher is the loss of coverage. Both figures are a representation of the monopartite event network related to infertility.

**Table S11 (sheet 11): Information loss for male anogenital distance** (See Supplementary tables: sheet11)

${H^{1}}_{\mathrm{network}}^{\mathrm{before}}$: initial uncertainty in term of network, ${H^{2}}_{\mathrm{nodes}}^{\mathrm{before}}$: initial uncertainty in term of nodes. Value of each H^before^ subgroup was averaged based on each node in given subgroup;${H^{3}}_{\mathrm{network}}^{\mathrm{after}}$: uncertainty after projection to a monopartite network; ${H^{4}}_{\mathrm{nodes}}^{\mathrm{after}}$: uncertainty score associated to nodes after projection to a monopartite network. Value of each subgroup was averaged on H^after^ for each node in given subgroup; $\Delta{H^{5}}_{\mathrm{network}}$: increase in uncertainty for network; $\Delta{H^{6}}_{\mathrm{nodes}}$: increase in uncertainty for nodes;${\neg\mathrm{COV}^{7}}_{\mathrm{edge}}$: loss of coverage at level of edge. The score for each subgroup considers all the edges that link nodes belonging to target subgroup. Then, the average of loss of coverage for each edge is calculated;${\neg\mathrm{COV}^{8}}_{\mathrm{node}}$: loss of coverage for nodes. Value of each subgroup was averaged based on each node in given subgroup to calculate the loss of coverage.

**References**

1. Schwartz CL, Christiansen S, Vinggaard AM, Axelstad M, Hass U, Svingen T. Anogenital distance as a toxicological or clinical marker for fetal androgen action and risk for reproductive disorders. Arch Toxicol (2019) **93**:253–272. doi:10.1007/s00204-018-2350-5

2. Mendiola J, Melgarejo M, Moñino‐García M, Cutillas‐Tolín A, Noguera‐Velasco JA, Torres‐Cantero AM. Is anogenital distance associated with semen quality in male partners of subfertile couples? Andrology (2015) **3**:672–676. doi:https://doi.org/10.1111/andr.12059

3. Eisenberg Michael L., Jensen Tina K., Walters R. Chanc, Skakkebaek Niels E., Lipshultz Larry I. The Relationship Between Anogenital Distance and Reproductive Hormone Levels in Adult Men. Journal of Urology (2012) **187**:594–598. doi:10.1016/j.juro.2011.10.041

4. Eisenberg ML, Hsieh MH, Walters RC, Krasnow R, Lipshultz LI. The Relationship between Anogenital Distance, Fatherhood, and Fertility in Adult Men. PLOS ONE (2011) **6**:e18973. doi:10.1371/journal.pone.0018973

5. Mogensen DM, Pihl MB, Skakkebæk NE, Andersen HR, Juul A, Kyhl HB, Swan S, Kristensen DM, Andersen MS, Lind DV, et al. Prenatal exposure to antifungal medication may change anogenital distance in male offspring: a preliminary study. Environmental Health (2017) **16**:68. doi:10.1186/s12940-017-0263-z

6. Fisher BG, Thankamony A, Hughes IA, Ong KK, Dunger DB, Acerini CL. Prenatal paracetamol exposure is associated with shorter anogenital distance in male infants. Human Reproduction (2016) **31**:2642–2650. doi:10.1093/humrep/dew196

7. Schwartz CL, Svingen T, Taxvig C, Christiansen S, Pedersen M, Frandsen HL, Gøttsche AF, Frederiksen KA, Johansson HKL, Vinggaard AM. The prostate cancer drug enzalutamide shortens anogenital distance in male rat offspring by blocking the androgen receptor. In Review (2019). doi:10.21203/rs.2.17827/v1
